# Advice to the Deaf, &c.

**Published:** 1842-04

**Authors:** 


### ART. XVIII.

1. *Advice to the Deaf. The present State of Aural Surgery, &c.* By JOHN HARRISON CURTIS, Esq.—*London*, 1841.

2. *The Cephaloscope, and its uses in the Discrimination of the Normal and Abnormal Sounds in the Organ of Hearing, &c.* By J. H. CURTIS, Esq.—*London*, 1842.

3. *Deafness successfully treated through the passages leading from the Throat to the Ear, &c.* By J. YEARSLEY, M.R.C.S.—*London*, 1839.

4. *Improved Methods of treating Diseases of the Ear, &c.* By J. YEARSLEY, M.R.C.S.—*London*, 1840.

5. *An Outline of the principal Diseases of the Ear and their proper Treatment, &c.* By J. YEARSLEY, &c.—*London*, 1841.

6. *On Deafness from Morbid Conditions of the Mucous Membrane of the Stomach, Throat, and Ear, &c.* By J. YEARSLEY, &c.—*London*, 1842.

A sense of duty to our readers has induced us to peruse the publications whose titles we have just transcribed; and we believe that the very character of these titles is sufficient to indicate what our painful task has enabled us to declare positively, that the works to which they belong are not calculated for the perusal of the professional but of the general reader. They are, indeed striking illustrations of that unbounded and irrepressible philanthropy which has so generally distinguished the professors who have taken the ear and its maladies under their special protection; every page teeming with unreserved disclosures of the benefits conferred by the authors on the afflicted, and no concealment being made as to the place where and the means how those still in affliction may obtain relief. We had long beheld with astonishment the efforts made in this patriotic cause by Mr. Curtis; and we confess, we had deemed him not only unrivalled but unrivallable, seeing he had consecrated to it no less than SIXTEEN publications (the list is before us) previously to the appearance of the two at the head of this notice. But when we recollect that full five and twenty years have been given by Mr. Curtis to those labours of love, (*Cephaloscope*, p. 100,) and that Mr. Yearsley has produced the four works above enumerated, besides one recently reviewed by us on stammering—all within little more than three years, we must confess that Mr. Curtis seems at last to have met his match, both in the fervour of his philanthropy and the fertility of his resources. We have even some doubts whether Mr. Yearsley has not already achieved a wider fame and a higher glory than Mr. Curtis, if we may judge from the testimonials which he has kindly appended to his works. In one of these now before us, we have two pages of unmixed eulogy of Mr. Yearsley, his books, and his practice, from half the newspapers in the United Kingdom, comprehending such unquestionable authorities as the Times, Examiner, Age, Dublin Mail, Western Times, Salisbury Herald, Old England, Dorset Chronicle, Bath Herald, Morning Chronicle, Atlas, Weekly Chronicle, Morning Herald, Gloucester Journal, Exeter Post, Wilts Herald, Statesman, Brighton Guardian, Worcester Journal, Morning Post, Felix Farley, Bucks Herald, &c. It will be seen that there are no literary, medical, or scientific journals in this list; a circumstance which, in the case of the medical press at least, is satisfactorily explained in regard to Mr. Curtis, (and no doubt in regard to Mr. Yearsley also,) by " the petty jealousies and rivalries of some of the members of the medical profession," and by " the attacks to which he has been subjected by his less fortunate brethren." (Ceph. pp. 101-2.) This is the old melancholy story, verified since the beginning of time, of Envy pursuing Merit as its shadow; another example of the " assidua eminentis fortunæ invidia;" another " modern instance" how

" Invidus alterius rebus macrescit opimis."

But we hope our present notice will be considered by Mr. Yearsley and Mr. Curtis as some amends for the cruel silence of the medical press.

It is a singular and, indeed, unaccountable circumstance, that, notwithstanding this identity of patriotic zeal in these two benevolent individuals, and this community of suffering from the neglect or malignity of an ungrateful profession, there should exist anything like enmity between themselves. But such is the melancholy fact! Men of more tumid livers than ourselves, might rejoice at this; the philosophic enquirer might explain the fact on the principle that each had the best possible means of judging of the other's motives and pretensions: we, in our innocence, contemplate it with wonder, and deplore it in silence. Mr. Yearsley is a great operator; he achieves miracles with his knife, his scissors, his catheter, and his air-pump, and the world know and appreciate his doings. Mr. Curtis is a man of milder mood; he deprecates such bloody deeds; he even shudders at Mr. Yearsley's gentle irrigations of the Eustachian tubes.

"Bold operations," says this tender-hearted man, " are often undertaken by men least qualified to conduct them with success, by way of speculation, as it were; hoping if, in spite of their incapacity, the matter should terminate well, to gain credit, practice, and wealth, by means of their good fortune. The public having begun to entertain correct opinions as to the real merits of catheterism, those who had profited by the vogue in which that operation was held, finding themselves under the necessity of providing a substitute for this fast-failing source of revenue, set up a great cry about the prevalence of throat-deafness, and about some totally new, and of course completely successful, operation for its cure, consisting of cutting away the uvula and tonsils; and it appears from the statement of one of these gentlemen, that a considerable number of persons were hopeful enough to submit to this operation, which had not even the merit of novelty. ......... Owing to the vigorous exposure made of the futility of this piece of originality, I believe it has not very extensively imposed upon the public; and therefore, as this, like most other supposed new remedies, is susceptible of an infinite number of applications, it was suddenly discovered to be an unfailing cure for stammering." (Advice, pp. 34-5.)

" A great outcry," says the more heroic operator of Sackville Street, " has been raised by aurists of the old school (for so I must call them) against all kinds of surgical operations in the treatment of deafness, and the fears of the public have been excited by the false statements and exaggerated language employed by these sticklers for acoustic oils, acrid ointments, pink, blue, and yellow, and irritating injections, which, according to my own experience, is worse than useless. But ignorant as these persons appear to be of any scientific practice of their profession, they are nevertheless self-witted enough to foresee, in the more general acknowledgment and adoption of operative measures, the rapid downfal and extinction of their own absurd and inefficient treatment. The anathemas promulgated by these antiquated aurists, in their daily advertisements, forcibly remind one of the scarecrow in the cornfield. In time the public, like the birds, will become sensible of the deception practised upon them." (On Deafness, pp. 53-4.)

Antiquated aurists! "This was the most unkindest cut of all!" And from a brother too! "*Et tu, Brute!*" It is well for Mr. Curtis that the same retrospect which discloses his antiquity reveals an approving conscience.

"Twenty-five years," he says, with just pride, "have elapsed since I commenced this line of practice; and I have every reason to be satisfied with what I have accomplished in that period. ...... I have been followed by many persons of talent not only in this country, but on the Continent, both by regular and *irregular* members of the profession," &c. (Cephaloscope, p. 100.)

And, after such provocation, we may surely pardon even a philosopher if he occasionally exhibits a little warmth, or even wings slily an envenomed arrow at his traducers.

"The interference of the legislature," he significantly says, "is also required to prevent the intrusion of any not duly qualified persons into this branch of medical practice; as well on account of the serious evils their ignorance may occasion to those who come under their treatment, as of the discouragement which they present to competent persons from entering into the same profession." (Advice, p. 56.)

But, alas, even envenomed arrows can be returned.

"Never," says Mr. Yearsley, M.R.C.S., "never consult an aurist who is not an educated and *diplomatised* surgeon!" (Outline, p. 62.) .... "It is too well known," he adds, in another place, "that the attention of such people is directed only to the discovery of the most successful method of *imposing on public credulity*. Indeed, a blind empiricism has, *up to this time*, characterized the *soi-disant* aurist; and I would as soon rely on the old woman's nostrum as on his." (Deafness, &c., p. 3.)

Matchless pair! What rivalry in well-doing! What tender care for the lieges! What regard for education! What devotedness to science! What horror of quackery!

........ Arcades ambo,
Et cantare pares et respondere parati.

It would be to join with his enemies in the work of depreciation, if we closed this notice without some special reference to Mr. Curtis's newest book, the *Cephaloscope*. This is the name of an instrument invented by Mr. Curtis for exploring the ear by auscultation. It is simply a stethoscope terminated by a hollow bowl of sufficient size to inclose the whole of the external ear. Mr. Curtis derives wonderful information from the use of this instrument. "If the nostril," he says, "on the opposite side is closed by a finger covered with a handkerchief, whilst the patient inspires forcibly through the other, *the rushing of the air into the cavity of the tympanum* will be very evident, if the tube [Eustachian] is perfect." (p. 71.) We venture to say, that neither Mr. Wharton Jones nor Mr. Toynbee is cognisant of this singular phenomenon of the spontaneous rushing of air into a shut cavity without any external force; but this comes of the non-concentration of pursuits on one organ. They study the pathology of the ear as a branch of general surgical science—but what of that?

"It is only," says Mr. Curtis, "by a close and undivided attention to any one branch of the medical sciences that we can hope to attain eminence, and to be possessed of that skill by which we may benefit our suffering fellow-creatures, and add to the store, at present far too scanty, of medical knowledge." (Cephaloscope, p. 99.)

And these are not words of course: no, Mr. Curtis has stamped them with an "eternal blazon" of truth, and hallowed them by the most heroic self-sacrifice. "That I may be enabled," he says, "to devote my time and opportunities more exclusively to that interesting subject,...... I have resolved to abandon the study and treatment of the diseases of the eye, and restrict myself to aural pathology." (*Ib.*) Unheard-of magnanimity! It is, no doubt, for want of this "exclusive devotion" that we have found it impossible to make the bowl of the new instrument to fit the inequalities of surface in the aural region so as to exclude the access of the air—a circumstance, as all auscultators know, essential to the appreciation of stethoscopic phenomena. Unquestionably the Fellows of the Royal Society must have met with no such difficulty, else they never would have conferred on its inventor a mark of honour we believe unexampled in the annals of that society. "I have presented the instrument," says Mr. Curtis, "to the Royal and several other learned societies, *which have accorded it their approbation*, and returned me their thanks." (Cephaloscope, p. 88.) But possibly these learned societies had in view, in their award, some of Mr. Curtis's other admirable inventions; such as his *musoton*, an instrument whereby he instantaneously "draws out and restores to its natural condition" the membrana tympani when morbidly "concavo-convex in an improper sense;" or, more probably, his *acoustic chair*, which is certainly one of the most astonishing inventions in this wonder-working age. We regret that our limits will only permit us to notice one or two of the important public services which this marvellous chair is capable of rendering to society; but we declare that if, after reading these announcements, brief as they are—and we shall give them in the author's own words—Sir Robert Peel should hesitate to appoint Mr. Curtis not merely Aurist to the Queen, but Auscultator-General to the Cabinet and the Horse-Guards, we shall withdraw such confidence in his government as we have hitherto given it.

"Some years since I invented an acoustic chair, by means of which, with its connecting pipes, a communication could be sent from Downing Street or the Horse Guards to the Mansion House in a minute and a half, and to every police or fire-brigade station in the metropolis in the course of a short time; so that necessary intelligence, in case of fire or other calamity, could be conveyed with surprising rapidity........ My acoustic chair is so constructed, that, by means of additional tubes, &c., the person seated in it may hear distinctly, while sitting perfectly at ease, whatever transpires in any apartment from which the pipes are carried to the chair......... This invention is further valuable, and superior to all other similar contrivances, as it requires no trouble or skill in the use of it, and is so perfectly simple in its application, that a child may employ it with as much facility and as effectually as an adult. It is, moreover, a very comfortable and elegant piece of furniture...... By means of tubes of sufficient length, this chair might be made to convey intelligence from St. James's to the Houses of Lords and Commons, and even from London to the Queen at Windsor." (Cephaloscope, pp. 47-8, 50.)

Professor Wheatstone's magnetic telegraph is nothing to this: we are surprised that the railway companies should not have given the preference to the acoustic chair. Is it yet too late?

